# 2D materials for solar fuels via artificial photosynthesis

**DOI:** 10.1093/nsr/nwab116

**Published:** 2021-07-02

**Authors:** Jian Du, Hao Yang, Licheng Sun

**Affiliations:** Institute of Natural Sciences, Westlake Institute for Advanced Study, China; Center of Artificial Photosynthesis for Solar Fuels, School of Science, Westlake University, China; Department of Chemistry, School of Engineering Sciences in Chemistry, Biotechnology and Health, KTH Royal Institute of Technology, Sweden; Institute of Natural Sciences, Westlake Institute for Advanced Study, China; Center of Artificial Photosynthesis for Solar Fuels, School of Science, Westlake University, China; Department of Chemistry, School of Engineering Sciences in Chemistry, Biotechnology and Health, KTH Royal Institute of Technology, Sweden; State Key Laboratory of Fine Chemicals, DUT-KTH Joint Education and Research Centre on Molecular Devices, Dalian University of Technology, China

## Abstract

To accelerate the chemical processes of water splitting, CO2 reduction and N2 fixation in artificial photosynthetic systems, various strategies to improve the properties of 2D materials with catalysts are highlighted in this perspective.

To create a globally carbon neutral society, sustainable energy systems to replace fossil fuels are needed urgently. Photosynthesis in nature converts CO_2_ and H_2_O into carbohydrates and O_2_ and is driven by solar energy via photosystem II (PSII) and photosystem I (PSI) [1]. Water oxidation occurring at the oxygen-evolution complex (OEC: CaMn_4_O_5_) of PSII is the primary source of protons and electrons for the reduction of CO_2_ by Ribulose-1,5-biphosphate carboxylate/oxygenase (RuBisCo). The protons and electrons produced in PSII are also utilized directly or indirectly in other enzyme catalysis reactions such as the H_2_ evolution reaction (HER) by hydrogenases (H_2_ase), N_2_-fixing reaction by nitrogenase (N_2_ase) or O_2_ reduction reaction by cytochrome *c* oxidase (CCO) (Fig. 1). Inspired by the function of photosynthesis, chemical processes including water splitting, CO_2_ reduction and N_2_ fixation can be replicated via artificial photosynthesis (AP).

**Figure 1.**
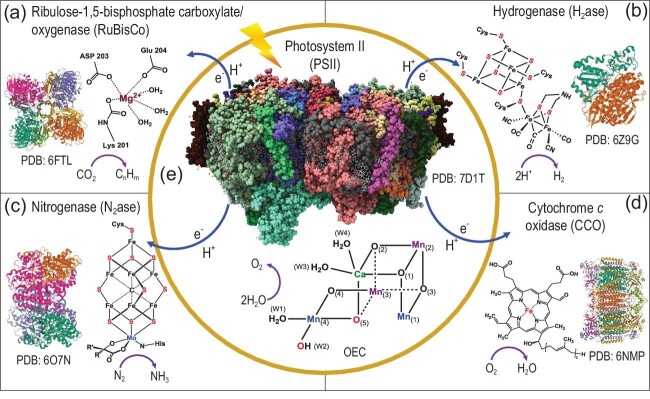
Schematic representation of biological enzymes and their active site structures and functions as potential inspiration for AP. (a) RuBisCo, (b) H_2_ase, (c) N_2_ase, (d) CCO and (e) PSII in nature.

Since the discovery of the first single-layer graphene (GR) in 2004 [2], numerous graphene-like two-dimensional (2D) materials including metal oxides/chalcogenides and metal-free materials (graphene derivatives, graphitic carbon nitride (g-C_3_N_4_), black phosphorus (BP), covalent-organic frameworks (COFs) etc.) (Fig. 2, top) have aroused widespread interest in artificial photosynthetic systems due to their unique physicochemical properties. 2D materials in AP systems can act as light absorbers to capture solar light, and simultaneously produce the high-energy state electron-hole pairs (Fig. 2, middle). Due to the merit of the nanoscale thickness of 2D materials, the photogenerated electrons can be swiftly conveyed to co-catalysts for the reduction reactions (HER, CO_2_ reduction, N_2_ fixation etc.), while the photogenerated holes are quickly transferred to water oxidation catalysts for O_2_ evolution reaction (OER). In addition to the rapid migration of photogenerated carriers, the large specific surface area of 2D materials can by themselves provide more accessible active sites, thus facilitating their catalytic activity. Furthermore, the planar structures of 2D materials allow their chemical modification via various strategies for AP [3].

From the viewpoint of morphology control, constructing 2D-material-based photocatalysts with various architectures is one of the efficient strategies to tune their properties. For example, 2D materials with a hollow sphere-like structure have exhibited remarkably improved catalytic activity toward water oxidation and CO_2_ reduction owing to the enhanced light absorption capability, efficient carrier separation and exposure of more active sites [4]. Although the assembly of 2D materials into a multi-level structure is beneficial for photocatalytic reactions, the poorly controlled interfacial engineering limits their further improvement. In this regard, reducing the thickness of 2D materials to atomic level is another efficient strategy to modulate their properties. This strategy can accelerate the electron transfer toward the in-plane direction and simultaneously reduce the recombination of photogenerated charge carriers [5]. Moreover, the ultrathin nature of 2D materials at atomic thickness causes the exposure of more interior atoms, and partial escape of these atoms from the lattice of ultrathin 2D layers gives rise to the formation of surface vacancies. It is widely accepted that the introduction of anionic or cationic vacancies can tune the band-position alignment for the improvement of light absorption. Moreover, the vacancy sites can act as active centers to capture photogenerated charges, therefore, the recombination of photogenerated electron-hole pairs could be efficiently inhibited [5]. However, the vacancies in some cases were also reported as recombination centers for the electron-hole pairs, resulting in the decreased density of carriers involved in photocatalytic reactions. This phenomenon may be caused by the formation of undesirable or excess vacancies that are detrimental to photocatalysis. Such a contradictory result makes the precise understanding of the relationship between vacancies and activity a challenge. Therefore, exploring efficient approaches to creating desirable vacancies with proper density in 2D materials is significant and strongly desired for highly efficient photocatalysts. Similar to the vacancy strategy, foreign elemental doping, including metal doping (Rh, Pt, Co etc.) and non-metal doping (C, B, N, P etc.), is also demonstrated to broaden the visible light absorption and simultaneously adjust the electronic structure of 2D photocatalysts via band alignment tuning [4].

**Figure 2.**
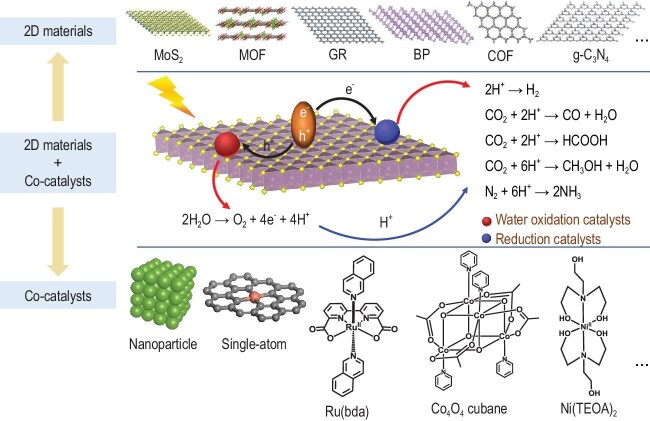
Integration of 2D materials and co-catalysts for AP.

Some 2D materials have shown excellent band compatibility, making it easy for them to interact with various dimensional nanomaterials to form heterostructures such as 0D/2D, 1D/2D, 2D/2D and 2D/3D composites [6]. Among them, the face-to-face 2D/2D nanostructures featuring large intimate contact interfaces possess abundant channels for charge transfer and trap, therefore leading to improved charge separation. Based on the heterostructures, the built-in electric fields generated on the heterointerfaces of two 2D semiconductors can further accelerate charge transfer. However, the complexity of heterostructures makes the precise understanding of the charge transfer mechanism and nature of active sites difficult. Consequently, the development of feasible *in-situ/operando* characterization techniques together with corresponding theoretical calculations is important.

Although the properties of 2D materials can be tuned through the above-mentioned strategies, the unfavorable charge recombination and sluggish kinetics of the surface reaction still limit their photocatalytic performance. Principally, co-catalysts loaded on 2D materials can serve as charge trap centers to extract electron/hole from bulk to surface and to accelerate surface catalytic kinetics. Therefore, co-catalyst loading can efficiently relieve the undesirable issues. In general, the co-catalysts can be classified into two categories: heterogenous and molecular catalysts (Fig. 2, bottom) [7]. Heterogenous catalysts such as metal/metal oxide nanoparticles (Pt, Ag, Au, Cu, NiO_x_, CoO_x_ etc.) have been widely applied in AP due to their high stability. However, the large fraction of inactive sites inside the bulk not only limits the overall photocatalytic activity, but also causes complexities in catalytic mechanism investigations. Hence, design of heterogenous catalysts with well-defined active sites and high atomic utilization is urgently required for high performance and clear catalytic mechanisms. Downsizing the metal/metal nanoparticles to the atomic level is feasible, and the resultant so-called single-atom catalysts with isolated metal sites anchored on 2D materials have been under the spotlight in recent years due to the advantages of well-defined active sites and high atomic utilization (up to 100%). In addition, single-atom catalysts can alter the selectivity of products in some reactions such as CO_2_ reduction. In view of these advantages, single-atom catalysts provide an ideal means of facilitating AP. Molecular catalysts, as the functional mimics of natural enzymes, have the advantages of metal-atom economy, clear active sites, easy tunability, high selectivity and better-understood catalytic mechanisms. In artificial photosynthetic systems, molecular-heterogenous hybrids were fabricated by the integration of highly active molecular catalysts with 2D materials through physical/chemical adsorption or chemical bonds. For example, g-C_3_N_4_ showed notably improved performance in photocatalytic water splitting and CO_2_ reduction when Ru, Co or Ni-based molecular catalysts were immobilized on the surface of carbon nitride [5,8,9]. Nevertheless, molecular catalysts often suffer from poor durability during long-term operation. The rational design of 2D materials/molecular catalysts with high stability will be a crucial point in future studies.

Currently, many breakthroughs are being made in promoting photocatalytic water splitting, CO_2_ reduction and N_2_ fixation properties via 2D-material-based AP under lab conditions, while industrial application is still in its infancy. The lack of facile and economic technologies for large-scale production of 2D materials with a precisely controllable layer number and atomic structure is a non-negligible factor. In addition, as for photocatalytic CO_2_ reduction, the products are mainly limited to C_1_ chemicals instead of the C_2+_ chemicals with higher energy density and market value [3]. In comparison with traditional fossil fuels, the economic advantages of the C_1_ carbon-based products obtained from photocatalytic CO_2_ reduction are inconspicuous. Another issue is the relatively low photocatalytic activity of N_2_ fixation (micromole or below) caused by the high N≇N triple bond energy, weak adsorption and activation of N_2_ on catalytic sites [10], which is far below the requirement for practical application. Therefore, the systematic optimization of 2D materials and co-catalysts is the cornerstone to achieving large-scale production of solar fuels via AP.
